# Candidate effectors for leaf rust resistance gene *Lr28* identified through transcriptome and *in-silico* analysis

**DOI:** 10.3389/fmicb.2023.1143703

**Published:** 2023-09-17

**Authors:** Pramod Prasad, Neelu Jain, Jyoti Chaudhary, Rajni Kant Thakur, Siddanna Savadi, Subhash Chander Bhardwaj, Om Prakash Gangwar, Charu Lata, Sneha Adhikari, Subodh Kumar, Harindra Singh Balyan, Pushpendra Kumar Gupta

**Affiliations:** ^1^ICAR-Indian Institute of Wheat and Barley Research, Regional Station, Shimla, India; ^2^Division of Genetics, ICAR-Indian Agricultural Research Institute (IARI), New Delhi, India; ^3^Department of Genetics and Plant Breeding, Chaudhary Charan Singh University, Meerut, India; ^4^ICAR-Directorate of Cashew Research, Puttur, India

**Keywords:** wheat, leaf rust, *Puccinia*, pathogenicity, Avr genes, qRT-PCR

## Abstract

*Puccinia* spp. causing rust diseases in wheat and other cereals secrete several specialized effector proteins into host cells. Characterization of these proteins and their interaction with host’s R proteins could greatly help to limit crop losses due to diseases. Prediction of effector proteins by combining the transcriptome analysis and multiple *in-silico* approaches is gaining importance in revealing the pathogenic mechanism. The present study involved identification of 13 *Puccinia triticina* (*Pt*) coding sequences (CDSs), through transcriptome analysis, that were differentially expressed during wheat-leaf rust interaction; and prediction of their effector like features using different *in-silico* tools. NCBI-BLAST and pathogen-host interaction BLAST (PHI-BLAST) tools were used to annotate and classify these sequences based on their most closely matched counterpart in both the databases. Homology between CDSs and the annotated sequences in the NCBI database ranged from 79 to 94% and with putative effectors of other plant pathogens in PHI-BLAST from 24.46 to 54.35%. Nine of the 13 CDSs had effector-like features according to EffectorP 3.0 (≥0.546 probability of these sequences to be effector). The qRT-PCR expression analysis revealed that the relative expression of all CDSs in compatible interaction (HD2329) was maximum at 11 days post inoculation (dpi) and that in incompatible interactions (HD2329 + *Lr28*) was maximum at 3 dpi in seven and 9 dpi in five CDSs. These results suggest that six CDSs (>0.8 effector probability as per EffectorP 3.0) could be considered as putative *Pt* effectors. The molecular docking and MD simulation analysis of these six CDSs suggested that candidate *Lr28* protein binds more strongly to candidate effector c14094_g1_i1 to form more stable complex than the remaining five. Further functional characterization of these six candidate effectors should prove useful for a better understanding of wheat-leaf rust interaction. In turn, this should facilitate effector-based leaf rust resistance breeding in wheat.

## Introduction

Leaf rust on wheat (*Triticum* sp) is caused by *Puccinia triticina* Erikss. & Henn. (*Pt*), an obligate biotroph, which is macrocyclic and heteroecious in nature. It causes yield losses in all wheat-growing areas of the world, sometimes approaching ~50% or more during epidemics (Bolton et al., 2008; Huerta-Espino et al., 2011; Prasad et al., 2017). The development and deployment of leaf rust resistant wheat cultivars is the most effective, economical and eco-friendly approach to manage wheat rusts. In wheat and related species, >220 resistance genes (*R* genes) for the three rusts have been identified; many of these *R* genes have also been utilized in resistance breeding in wheat (McIntosh et al., 2017; Prasad et al., 2019; Kumar et al., 2021). Among these genes, 11 stem rust (*Sr*), seven yellow rust (*Yr*), and six leaf rust (*Lr*) resistance genes have also been cloned and characterized (Thind et al., 2017; Prasad et al., 2019, 2020; Luo et al., 2021).

In recent years, the study of molecular basis (including epigenomics) underlying host-pathogen interactions has been a priority area of research (Kamoun, 2007; Hogenhout et al., 2009; Savadi et al., 2017a,b; Prasad et al., 2018). As a result, it is now known that wheat-rust interaction is regulated by multilayered immune system in a coordinated manner, which follows Flor’s gene-for-gene hypothesis (Flor, 1955). The two main layers of plant immunity include the PTI (PAMP triggered immunity) and the ETI (effector triggered immunity) (Jones and Dangl, 2006; Vleeshouwers and Oliver, 2014). Keeping this in view, efforts are being made to identify Avr genes and the effector molecules of the pathogen and the corresponding *R* gene of the host (Walton et al., 2009; Vleeshouwers and Oliver, 2014; Prasad et al., 2019; Wei et al., 2020).

The genome sequences are now available for a large number of pathogens. The availability of robust statistical/bioinformatics tools also facilitated identification of the potential effectors that are also described as ‘candidate secreted effector proteins’ (CSEPs) including those belonging to the three wheat rusts (Upadhyaya et al., 2015; Kiran et al., 2016, 2017; Cuomo et al., 2017). The secretory proteins encoded by the Avr genes lack conserved sequences and therefore, their prediction is challenging. Under these conditions, the identification of effectors is primarily based on characteristic features like presence of a secretion signal, small size (<300 amino acids) and cysteine rich proteins (Sperschneider et al., 2015; Sonah et al., 2016). Several *in silico* tools have also been developed for prediction of effectors (Sonah et al., 2016; Prasad et al., 2019). This has led to a new research area called ‘effectoromics’ (Sonah et al., 2016).

The efficiency of effector prediction is improving with the availability of fungal secretomes and the following known parameters of the effector molecules: molecular weight, the net charge, and the occurrence (per cent) of cysteine, serine, and tryptophan residues (Sperschneider et al., 2018). For identification of candidate effectors several approaches have been used. An effective approach is to identify pathogen secretions directly using proteomics approaches or alternatively, transcriptome analysis of infected tissues can be used to identify the putative transcripts followed by bioinformatics analysis (Jaswal et al., 2019; Prasad et al., 2019). RNA-seq also allows identification of transcripts that have low expression levels or occur in low frequencies. Among the known Avr genes for wheat rusts, only *AvrSr27* (Upadhyaya et al., 2021), *AvrSr35* (Salcedo et al., 2017), and *AvrSr50* (Chen et al., 2017) for stem rust have been characterized; no Avr gene and/or effector molecule for *Pt* has been characterized. However, several candidate effectors/Avr genes have been suggested for *Pt*: Pt77192, Pt5974, Pt34354, Pt23713, Pt1625, and Pt36553 (Zhang et al., 2020); PTTG_08198 (Zhao et al., 2020); PTTG_25509 (*AvrLr24* candidate) (Bruce et al., 2014); PtMAPK1, PtCYC1, and PtCNB (Panwar et al., 2013a,b); Pt3 and Pt27 (Segovia et al., 2016); *AvrLr20* (Wu et al., 2017); *AvrLr1*, *AvrLr15*, and *AvrLr24* (Song et al., 2020).

In the present study, using transcriptome data generated in our earlier study, we identified 13 *Pt* coding sequences (CDSs) having the features of effectors (Sharma et al., 2018) and validated their expressions during the incompatible (HD2329 + *Lr28*) and compatible (HD2329) interactions in a pair of near-isogenic lines (NILs) at different time points after inoculation. We also carried out docking and MD simulation analysis involving candidate *Lr28* protein and candidate effector. To the best of our knowledge, this is the first effort to identify a candidate effector for the gene *Lr28*, which is an effective *R* gene that has been utilized in several resistance breeding programs in India.

## Materials and methods

### Identification of pathogen transcripts secreted during interaction with host

**Figure 1:**
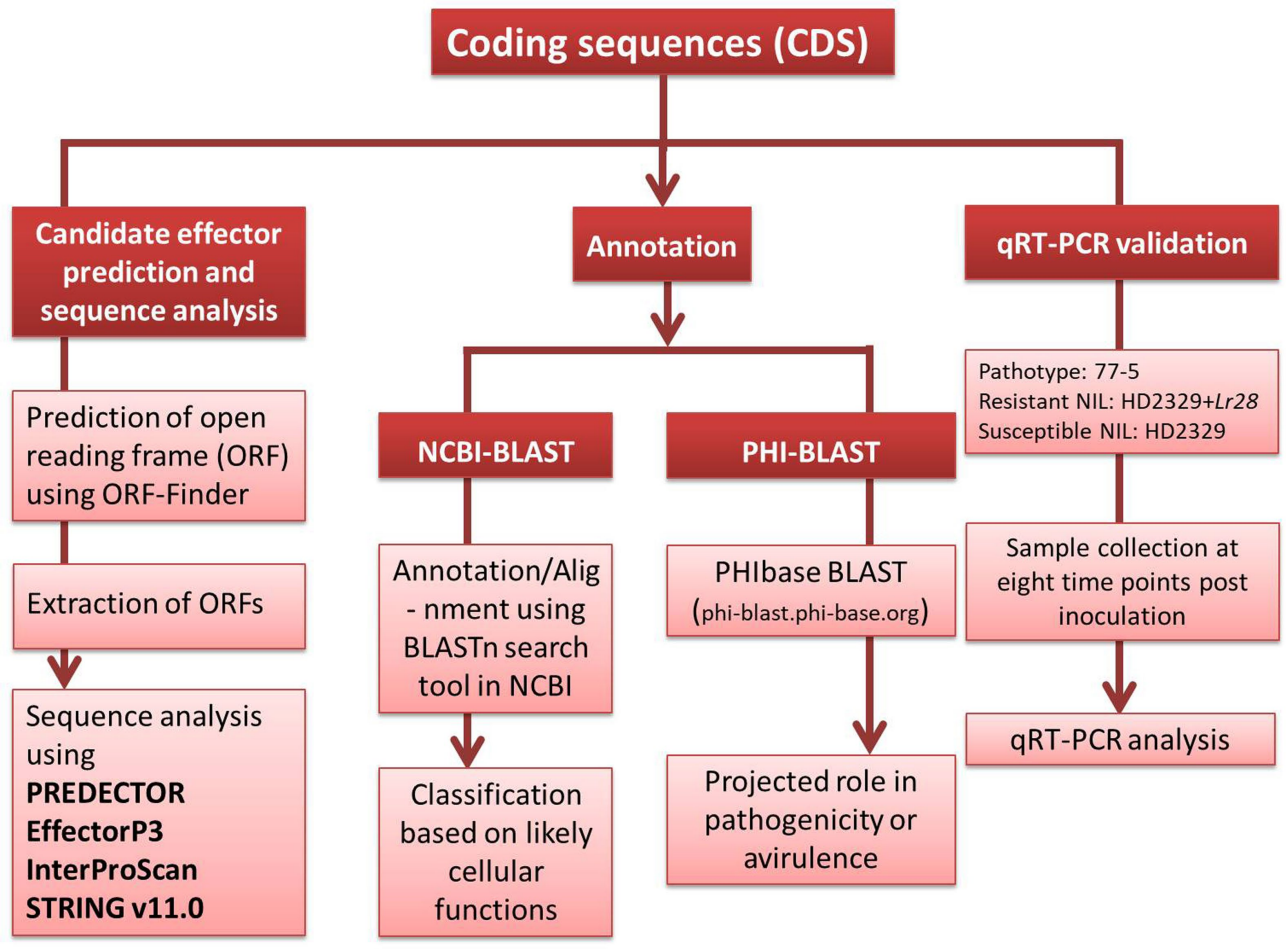
The methodology pipeline adopted to study *P. triticina* coding sequences.

The detailed methodology for wheat-leaf rust transcriptome analysis of infected leaf tissues, including RNA isolation, RNA sequencing, bioinformatics analysis of RNA-sequence data etc. is available in our earlier report (Sharma et al., 2018). Briefly, for identification of host genes, the raw sequence reads were aligned to four reference genomes in the following order: draft genome of *T. aestivum* L. (82.21%), *Aegilops tauschii* Coss. (2.89%), *T. urartu* Thumanjan ex Gandilyan (3.02%) and *Brachypodium distachyon* (0.003%), and wheat expressed sequence tags (ESTs) database (6.1%) downloaded from http://www.ncbi.nlm.nih.gov/nucest/?term=Triticum+aestivum. The remaining 5.77% unaligned reads (after alignment with the above four reference genomes and wheat ESTs database) were used for the identification of pathogen sequences. These unaligned reads were assembled *de novo* using Trinity with default options. The *de novo* assembled transcripts were annotated using Contig annotator pipeline. The *de novo* assembled transcripts were further searched for homologies with pathogen sequences in NCBI non-redundant protein database (accessed on June, 2018) using Blastx program with cut off 1e-50. Blastx analysis revealed a total of 13,381 transcripts (0.24%) that matched the genome sequences of *Puccinia* species [13,350 transcripts belonged to *P. graminis* f. sp. *tritici* (*Pgt*), 20 to *P. striiformis* f. sp. *tritici* (*Pst*), and 11 to *Pt*]. All the transcripts (13381) belonging to the pathogen were searched for coding sequences (CDSs) using NCBI database. Thirteen of these sequences, whose expressions differed between resistant and susceptible NILs (Supplementary file S1), were selected and classified according to their likely cellular functions (Table 1) and deposited in the NCBI database (Accession numbers MW423354 to MW423366). A brief methodology pipeline adopted to study *Pt* CDSs is outlined in Figure 1.

**Table 1:** Thirteen coding sequences from *P. triticina* showing significant homology to known fungal pathogenicity factors/proteins.

Sequence ID (accession number)	*Puccinia graminis* f. sp. *tritici*			*Puccinia triticina*		
	Annotation	Homology with sequences in NCBI GenBank (accession number)*	Identity (%) and query cover (%)	Annotation	Homology with sequences in NCBI GenBank (accession number)**	Identity (%) and query cover (%)
c45137_g1_i1 (MW423354)	Stress-induced-phosphoprotein 1	M_003324883	88 and 71	Uncharacterized protein	XM_053163789	100 and 69
c55520_g1_i2 (MW423355)	Acetyl-CoA C-acetyltransferase	XM_003335520	84 and 78	Uncharacterized protein	XM_053164692	99.92 and 79
c86066_g1_i1 (MW423356)	Sterol 24-C-methyltransferase	XM_003335761	87 and 66	Uncharacterized protein	XM_053166448	100 and 65
c21850_g1_i2 (MW423357)	Hypothetical protein	XM_003327581	87 and 49	Uncharacterized protein	XM_053161138	100 and 54
c12139_g1_i1 (MW423358)	60S ribosomal protein L42	XM_003328147	94 and 61	Uncharacterized protein	XM_053162181	99.5 and 51
c10109_g1_i2 (MW423359)	60S ribosomal protein L7	XM_003321171	91 and 78	Uncharacterized protein	XM_053160252	100 and 78
c40637_g1_i2 (MW423360)	40S ribosomal protein S5	XM_003324356	93 and 78	Uncharacterized protein	XM_053163940	99.6 and 75
c3093_g1_i2 (MW423361)	Hypothetical protein	XM_003325788	79 and 72	Uncharacterized protein	XM_053169090	100 and 74
c14094_g1_i1 (MW423362)	60S ribosomal protein L23	XM_003327862	91.6 and 68	Uncharacterized protein	XM_053166860	100 and 58
c48436_g1_i1 (MW423366)	40S ribosomal protein S27	XM_003337046	89 and 75	Uncharacterized protein	XM_053170220	100 and 39
c12596_g1_i2 (MW423363)	Hypothetical protein	XM_003333256	87 and 74	Uncharacterized protein	XM_053172629	100 and 72
c43030_g2_i1 (MW423364)	Hypothetical protein	XM_003335687	79 and 8	Chromosome 17B	CP110455	99.4 and 100
c13574_g1_i1 (MW423365)	Seryl-tRNA synthetase	XM_003319200	85.3 and 90	Uncharacterized protein (PtA15_10A405)	XM_053160577	100 and 89

Accession numbers belong to *Puccinia graminis f. sp. *tritici* CRL race 75-36-700-3; **Puccinia triticina race PtA15.

For submitting the above sequences to NCBI, the “CDS feature” was selected in the BLAST hits to get the CDS region in the alignment, which was followed by standard CDSs submission procedure. In one sequence (c48436_g1_i1) there were many deletions and/or insertions which caused reading frame shift. In order to deal with this, the frame-shifted sequences were submitted, with a miscellaneous feature, instead of a “Coding Region (CDS)/Gene/mRNA.” Similarly, in case of poor BLAST alignment across entire sequences, ORF finder^1^ was used to predict the coding region in the mRNA sequence, which was confirmed with protein BLAST search.

### Candidate effector prediction

The open reading frames (ORFs) for each of the 13 coding sequences of *Pt* were obtained through ORF-Finder in NCBI (see text footnote 1). The ORFs were utilized for obtaining the structure of corresponding proteins, which were in turn used to predict candidate effectors using the software EffectorP v3.0 (Sperschneider and Dodds, 2022) and PREDECTOR (Jones et al., 2021). PREDECTOR, a new pipeline that aggregates relevant features of fungal effector proteins utilizing multiple software tools and methods, was used for ranking of predicted effector candidates. The functional analysis of proteins was carried out using InterProScan (Blum et al., 2021) by classifying them into families, predicting domains and important sites. The search Tool for the Retrieval of Interacting Genes/Proteins (STRING v11.0) (Szklarczyk et al., 2019), a database of known and predicted protein-protein interactions, was used to predict the structure, domains and functions of nine *Pt* CDSs, which were predicted to have effector features using EffectorP v3.0. The 13 CDSs were also used for the identification of homologs of functionally characterized candidate effectors from other pathogens using PHI-BLAST^2^ (Urban et al., 2020).

### 3D-structures of receptor R protein and candidate effector molecules

Homology modeling was utilized to generate 3D structures of the candidate effectors which had >0.8 effector probability, and an anticipated *Lr28* protein (Sharma et al., 2018). The template structures were used to model 3D structures of the proteins (R protein + candidate effectors) using automated SWISS-MODEL server (Waterhouse et al., 2018) and template library following Kumar et al. (2016). The 3D structures were then visualized in different coordinates using UCSF CHIMERA 1.16 (Pettersen et al., 2004). The predicted 3D structures were verified by both geometric and energetic means using Structure Analysis and Verification Server (SAVES).^3^

### Quality assessment of 3D structures

The quality of 3D structures were checked using following three parameters: (i) the relative proportion of the number of amino acids, which fall in favored region, relative to other regions, using PROCHECK^4^ (Laskowski et al., 1993), (ii) the compatibility of the atomic model (3D) with its own amino acid sequence using VERIFY3D^5^ (Eisenberg et al., 1997), and (iii) analysis of the statistics of non-bonded interactions between different atom types, using ERRAT^6^ (Colovos and Yeates, 1993).

### Molecular docking analysis

The molecular docking was performed using ClusPro protein-protein docking server^7^ (Kozakov et al., 2017; Vajda et al., 2017; Desta et al., 2020). The docking results were visualized in the PyMOL^8^ (Schrödinger and DeLano, 2020) to confirm the binding position of R protein and the candidate effectors. Other interaction graphics were generated using the EMBL-EBI tool PDBsum.^9^ The values for binding affinity (ΔG) and dissociation constant (KD) were obtained from the Prodigy server^10^ (Vangone and Bonvin, 2015; Xue et al., 2016). The UCSF Chimera package 1.16 (Pettersen et al., 2004) was used to display the position of the interaction between R protein and candidate effectors.

### Molecular dynamics simulation

The dynamic behavior of candidate effectors with R protein lineage complexes was studied by MD simulation performed on GROMACS^11^ (Case et al., 2018), using the charm27 protein force field. The solvation of the system was done using the TIP3P water model with a margin of 10 Å (Jorgensen et al., 1983; Maier et al., 2015). Following this, the system was neutralized by the addition of counter ions. Then, two consecutive minimization steps were performed before the long MD simulation. In the first, 3,000 iterations (1,000 steepest descents and following 2,000 conjugate gradients) were submitted. In the second minimization step, 4,000 iterations (2,000 steepest descents and following 2,000 conjugate gradients) were performed. In the first minimization step, atom coordinates for the whole system were restrained to their initial coordinates with a force constant of 10 kcal/mol Å − 2. In the second minimization step, the whole system was freely minimized to relieve atomic clashes/contacts in the entire system. The system was then heated at 300 K through 100 ps of MD with a time step of 2 fs per step, while the whole system was restrained again with a force constant of 10 kcal mol^−1^ Å^−2^. This was followed by density evaluation of the system through 100 ps of MD with a time step of 2 fs per step. Before the long MD simulation, an equilibration step (a short MD for 200 ps) was carried out to equilibrate the system. Finally, a routine MD simulation for 20 ns was applied during which the temperature was kept at 300 K. The RMSD was calculated to predict the protein and protein-protein complex stability. For the calculation of RMSD, protein backbone was considered. The RMSF was also measured for predicting the flexibility of each amino acid residue after effector binding. The plots were generated by xmgrace plotting program.

### Host miRNA interaction with pathogen CDSs

The *Pt* CDSs were examined as potential targets of host miRNA using the psRNATarget web server (Dai et al., 2018)^12^ with the default parameters and maximum expect value of 3. For this purpose, both *in-silico* and deep sequencing data for known and novel miRNA were available from our earlier studies (Jain et al., 2020, 2023).

### Expression profiling of Pt CDSs

#### Inoculation of wheat seedlings and sample collection

Seedlings of a pair of NILs (HD2329; susceptible and HD2329 + *Lr28*; resistant) were grown under controlled conditions of 16 h light at 25°C and 8 h dark at 18°C in a greenhouse. The fully expanded primary leaves of these plants were spray-inoculated with uredospores suspension (50 mg/mL) of *Pt* pathotype 77–5 {121R63-1 (Indian binomial notation), THTTM (North American Notation)} in non-phytotoxic isoparaffinic mineral oil soltrol (Chevron Phillips Chemical Company, United States) following Prasad et al. (2018). The inoculated plants, after 48 h of incubation in high humidity chambers, were transferred to separate glass house chambers maintained at 22 ± 2°C and > 80% relative humidity. Leaf samples for RNA isolation were collected in triplicates at eight time points (1, 2, 3, 4, 5, 6, 9, and 11 days post inoculation, dpi), frozen in liquid nitrogen, and stored at −80°C until RNA isolation.

**Figure 2:**
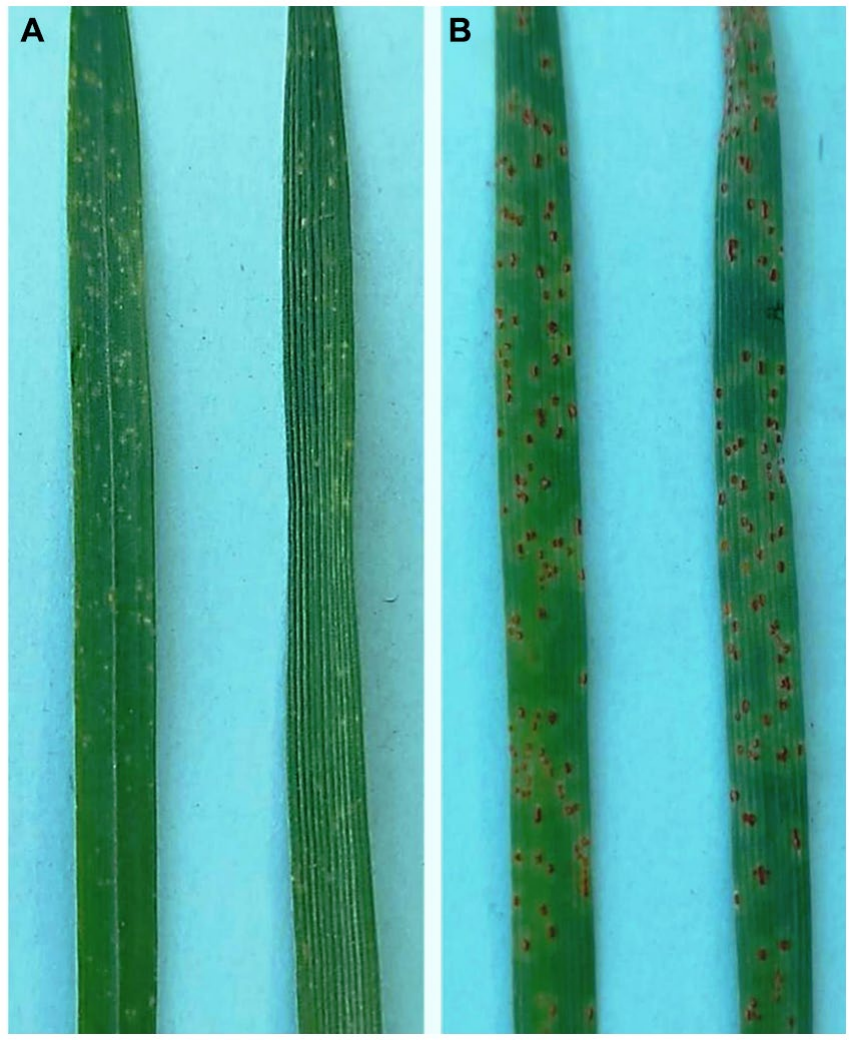
Phenotypic response of **(A)** resistant (HD2329+ *Lr28*) and **(B)** susceptible (HD2329) NILs after 14 days of inoculation with *P. triticina* pathotype 77-5.

Leaf rust infection types were scored using the rust infection scale of Stakman et al. (1962) with slight modifications. The initial symptoms of the leaf rust (as flecking) were observed 5 dpi on HD2329 (susceptible NIL). The uredia as raised pustules, corresponding to 3+ to 4 infection type (susceptible), were fully developed on 14th day after inoculation on susceptible NIL (HD2329), whereas the plants of resistant NIL (HD2329 + *Lr28*) remained disease free (infection type: ; or 0;) (Figures 2A,B).

#### RNA isolation and cDNA preparation

The leaf tissue (100 mg) was homogenized in a FastPrep^®^-24 tissue lyzer (MP Biomedicals, USA). RNA was isolated from homogenate using the QIAGEN RNeasy Mini Kit (Qiagen, Germany) according to the manufacturer’s instructions. The integrity, yield, and purity of total RNA was determined using 1.4% formaldehyde gel electrophoresis (Rio, 2015) followed by estimation of concentration using NanoDrop 2000c^®^ UV-Vis Spectrophotometer (Thermo Scientific, United States). The cDNA was prepared from 2 μg of total RNA using a High-Capacity cDNA Reverse Transcription Kit with Oligo (dT) primer (Applied Biosystems, United States) following the prescribed protocol.

#### qRT-PCR and data analysis

The qRT-PCR primers for all the 13 *Pt* coding sequences and reference genes were designed using the Primer Express Software v3.0.1 and synthesized from Imperial Life Science (Gurugram, India). The amplification efficiency (E) of each primer was calculated following Bustin et al. (2009).

Three *Pt* reference genes, namely ‘Glyceraldehyde-3-Phosphate Dehydrogenase (GAPDH), Tubulin (TUB), and Actin (ACT) (Table 2) were used for their stable expression during the whole infection process and to normalize the expression data of all the 13 *Pt* coding sequences. The stability of different reference genes was analyzed using NormFinder and BestKeeper programmes (Andersen et al., 2004; Pfaffl et al., 2004). Actin gene with lowest standard deviation (SD; 0.91) and coefficient of variation (CV; 2.47) values in BestKeeper and lowest stability value (0.015) in NormFinder (Table 3), was found to be the best reference gene and therefore, selected for undertaking qRT-PCR analysis of *Pt* CDSs.

**Table 2:** Sequences, amplicon size, and amplification efficiency of primers used for qRT-PCR.

S No.	Sequence ID	Primer name	Sequence (5′-3′)	Amplicon size (bp)	Amplification efficiency (%)
**Coding sequences**					
1	c45137_g1_i1	RTPt_5	F: AACATCGAACCCCTGACACC	97	92
			R: TCGGAAAGCTCTGGGTTGAT		
2	c55520_g1_i2	RTPt_6	F: GATGACCATCGCTGAAGCAG	98	101
			R: GTCGAATCGTCGTCAGCTTG		
3	c86066_g1_i1	RTPt_7	F: AATGGTGCATGACGGACAAG	95	102
			R: CCTCATTTCGGCAATTCCAT		
4	c21850_g1_i2	RTPt_10	F: ATGGAGAGCACGCGGTTATT	101	98
			R: ATTCCTGGGCTAGCATTCCA		
5	c12139_g1_i1	RTPt_11	F: CCCCCACAAGGTCACTCAAT	95	99
			R: GTCCACCGTAACCGGATTGT		
6	c10109_g1_i2	RTPt_14	F: ATGACCTGACGCTTGGCTTT	100	102
			R: CTTTGCTGAAGAAGCGCAAA		
7	c40637_g1_i2	RTPt_15	F: TCAACTCGTCGGCAAGACAT	107	102
			R: CCCCTTACGACGAGTCAACC		
8	c3093_g1_i2	RTPt_16	F: CAATGGAAGTGGCCTGCATA	100	99
			R: ACCGGCACCTTCTTCCTTTT		
9	c14094_g1_i1	RTPt_18	F: ATTCTTGGCACCCGAGTTGT	97	96
			R: GTCGAACAAGGCCAAGGCTA		
10	c48436_g1_i1	RTPt_20	F: CCTTCGGTCAGACGAGCTTT	95	102
			R: ACCGTCTTCTCACACGCTCA		
11	c12596_g1_i2	RTPt_21	F: TCGGACCTATGGGTTTGGTC	107	103
			R: CCCGAGACCTCTTGAGCAGT		
12	c43030_g2_i1	RTPt_22	F: CGAAGACTGCGAGGATTGTG	98	98
			R: CCATTACCCCATTCCCCTCT		
13	c13574_g1_i1	RTPt_28	F: TCGGCTTCTTCCTTTTCCAA	107	97
			R: AGAATCGGTGAGGCCAAAAA		
Housekeeping genes (HKGs)*					
		RT-GAPDH	F: CAACGCTAGCTGCACCACTA	161	96
			R: TTCCACCTCTCCAGTCCTTG		
		RT-TUB	F: CCGATCAATTCACGGCCATGTTCA	174	92
			R: AACCCTCTTCAACTTCCTCGTCGT		
		RT-ACT	F: TGTCGGGTGGAACGACCATGTATT	146	100
			R: AGCCAAGATAGAACCACCGATCCA		

*Yin et al. (2009).

**Table 3:** Stability analysis of candidate reference genes based on BestKeeper and NormFinder.

Analysis/data	Reference genes		
	GAPDH	TUB	ACT
**BestKeeper analysis**			
std dev [±CP]*	1.92	1.61	0.91
CV [% CP]	6.01	4.56	2.47
**NormFinder analysis**			
Stability value	0.068	0.068	0.015

*std dev, Standard Deviation; CV, Coefficient of variation; CP, capability potential.

The qRT-PCR reaction mixture and thermal profile were as described by Prasad et al. (2018). The relative transcript levels of these sequences were quantified following the comparative Ct (ΔΔCt) method (Schmittgen and Livak, 2008) in Bio-Rad CFX Manager v3.1 software. Standard deviation between the cycle threshold values was also calculated using Bio-Rad CFX Manager v3.1. Tukey’s *post hoc* tests were used to determine the statistical difference among relative expression profiles of all CDSs.

## Results

### Identification and alignment of CDSs of the pathogen

The pathogen sequences (13381) were annotated using the BLASTn search tool in NCBI and the best match was available for 13 *Pt* coding sequences with *Pt* race PtA15 and *Pgt* race CRL 75-36-700-3. Significant homology was observed between sequences generated by us and the annotated sequences in the database; the sequence identity ranged from 94.4 to 100 and 79 to 94% with *Pt* and *Pgt* races, respectively, while query cover ranged between 39 to 100 and 8 to 78% with *Pt* and *Pgt* races, respectively (Table 1). Based on annotation, the 13 CDSs belonged to 10 categories of annotated sequences of *Pgt* race CRL 75-36-700-3, each category having only one sequence except for the category of a hypothetical protein, which had four sequences (see Table 1). The highest sequence identify of all CDSs was with the sequences of uncharacterized proteins of *Pt* race PtA15.

### Candidate effector prediction

**Figure 3:**
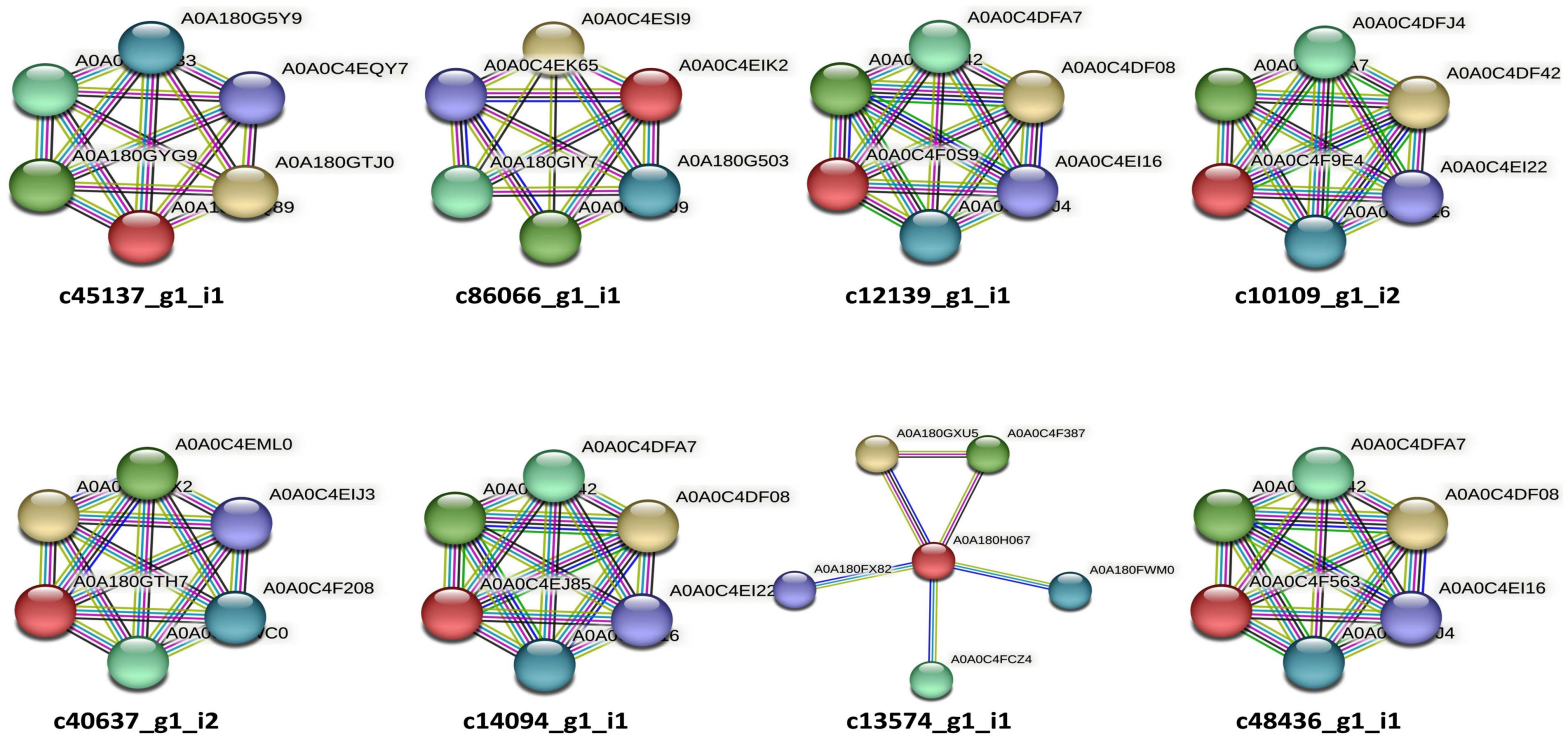
STRING (v11.0) analysis showing the predicted molecular action among the predicted candidate effectors of *P. triticina*. The gene nomenclatures of these interactions are depicted in Table 5. Network nodes represent different genes, while lines of different colors represent different types of evidences used in predicting associations of candidate effectors with these genes.

EffectorP analysis revealed that nine of the 13 coding sequences could be probable effectors, with their probability ranging from 0.546 (c86066_g1_i1) to 0.986 (c12139_g1_i1). All the predicted effectors were cytoplasmic except c48436_g1_i1, which was predicted to have both cytoplasmic (0.889) and apoplastic (0.768) features. The effector probability of the following six sequences was >0.8: c12596_g1_i2, c14094_g1_i1, c10109_g1_i2, c48436_g1_i1, c40637_g1_i2, and c12139_g1_i1 (Table 4). EffectorP analysis using PREDECTOR pipeline also validated these findings. The manual effector score in PREDECTOR analysis ranged between-7.288 (c55520_g1_i2) to 4.98 (c14094_g1_i1) (Table 4). Similarly, TMHHM score for first 60 amino acids ranged up to 24.46 (c14094_g1_i1). The coding sequence c43030_g2_i1 was ranked as the best candidate for effector in PREDECTOR analysis; however, it was not predicted to be effector in EffectorP, STRING and InterProScan analysis. Significant homology was observed between eight effector candidates and the sequences available in STRING database and InterProScan, however no similarity was found for c12596_g1_i2 in both STRING and InterProScan databases (Table 5). Among the eight effector candidates in STRING database, the predicted functional partners for three CDSs, namely c45137_g1_i1, c86066_g1_i1, and c13574_g1_i1 were heat shock protein, fatty acid hydroxylase domain-containing protein, and threonyl-tRNA synthetase known for helping in protein folding, ergosterol biosynthetic process, and mitochondrial seryl-tRNA aminoacylation, respectively. Similar domains and functions were predicted for these three sequences in InterProScan searches. The remaining five sequences (c12139_g1_i1, c10109_g1_i2, c40637_g1_i2, c14094_g1_i1, and c48436_g1_i1) were predicted to be ribosomal proteins in both STRING and InterProScan searches, which corroborated with NCBI BLAST. The results of STRING and InterProScan searches are summarized in Table 5 and Figure 3.

**Table 4:** A summary of PREDECTOR and EffectorP3 analysis for effector prediction and ranking statistics of CDSs of *P. triticina.*

Sequence ID	Effector score	Manual effector score	TMHMM (First 60)	Effector probability						
				EffectorP3 (cytoplasmic)	EffectorP3 (apoplastic)	ApoplastP	Signalp3_HMM	TargetP	SignalP3_nn	SignalP5
c43030_g2_i1	0.24	2.234	29.46	0	0	0.27	1	1	0.924	0.981
c14094_g1_i1	−0.916	4.981	0.48	0.856	0	0.56	1	0.132	0.345	0.062
c55520_g1_i2	−1.092	−7.288	0	0	0	0.15	0	0	0.155	0.001
c48436_g1_i1	−1.431	2.638	1.2	0.889	0.768	0.58	0	0.004	0.043	0.002
c12139_g1_i1	−1.874	−0.377	0	0.986	0	0.48	0	0	0.055	0.002
c10109_g1_i2	−2.057	−2.915	0	0.857	0	0.13	0	0	0.118	0
c40637_g1_i2	−2.122	1.591	0	0.915	0	0.13	0	0	0.1	0.001
c3093_g1_i2	−2.769	−6.685	0	0	0	0.5	0	0	0.031	0.001
c86066_g1_i1	−3.057	−2.228	0	0.546	0	0.12	0	0.001	0.038	0.002
c21850_g1_i2	−3.061	−6.738	8.99	0	0	0.08	0	0.178	0.74	0.029
c45137_g1_i1	−3.346	−0.433	0	0.788	0	0.12	0	0	0.023	0.001
c13574_g1_i1	−3.346	−3.199	0	0.688	0	0.03	0	0	0.036	0.001
c12596_g1_i2	−3.371	−2.238	0.47	0.811	0	0.24	0	0.001	0.298	0.001

**Table 5:** A summary of STRING and InterProScan search analysis of predicted candidate effectors of *P. triticina.*

Sequence ID	STRING					InterProScan		
	Target organism (Race)	Predicted functional partners	Homology score	Biological process (gene ontology)	KEGG pathway	Protein family membership	Biological process	Molecular function
c45137_g1_i1	*Puccinia triticina* 1-1 BBBD Race 1	Heat shock protein 90-1	0.995	Protein folding	Protein processing in endoplasmic reticulum	Heat shock protein STI1-like	None	Protein binding
c86066_g1_i1	*Puccinia triticina* 1-1 BBBD Race 1	Fatty acid hydroxylase domain-containing protein	0.874	Ergosterol biosynthetic process	Steroid biosynthesis	SAM-dependent methyltransferase Erg6/SMT-type	Steroid biosynthetic process	Methyltransferase activity
c12139_g1_i1	*Puccinia triticina* 1-1 BBBD Race 1	60S ribosomal protein L32	0.999	Cytoplasmic translation	Ribosome	Ribosomal protein L44e	Translation	Structural constituent of ribosome
c10109_g1_i2	*Puccinia triticina* 1-1 BBBD Race 1	50S ribosomal protein L22	0.999	Cytoplasmic translation	Ribosome	Ribosomal protein L7/L30	Maturation of LSU-rRNA from tricistronic rRNA transcript	Structural constituent of ribosome
c40637_g1_i2	*Puccinia triticina* 1-1 BBBD Race 1	40S ribosomal protein S18	0.994	Ribosomal small subunit assembly	Ribosome	Ribosomal protein S5/S7	Translation	Structural constituent of ribosome
c14094_g1_i1	*Puccinia triticina* 1-1 BBBD Race 1	60S ribosomal protein L32	0.999	Cytoplasmic translation	Ribosome	Ribosomal protein L14P	Translation	Structural constituent of ribosome
c48436_g1_i1	*Puccinia triticina* 1-1 BBBD Race 1	Ribosomal protein L15	0.999	Ribosomal small subunit assembly	Ribosome	Ribosomal protein S27	Translation	Structural constituent of ribosome
c12596_g1_i2	No similarity found	–	–	–	–	–	–	–
c13574_g1_i1	*Puccinia triticina* 1-1 BBBD Race 1	Threonyl-tRNA synthetase	0.982	Mitochondrial seryl-trna aminoacylation	Aminoacyl-tRNA biosynthesis	Serine-tRNA ligase, type1	seryl-tRNA aminoacylation	serine-tRNA ligase activity

The “PHI-base BLAST” results confirmed significant homology of *Pt* coding sequences with already known fungal putative effectors. The identities of these sequences ranged from 24.46 (c45137_g1_i1) to 54.35 (c3093_g1_i2) in PHI-base BLAST analysis. Each of 13 *Pt* CDSs had homology with putative effectors of one of the the following pathogens: *Phytophthora infestans* (potato), *Magnaporthe oryzae* (rice), *Bipolaris maydis* (maize), *Fusarium proliferatum* (rice), *Ustilago hordei* (barley), *Alternaria brassicicola* (Brassica), *Fusarium graminearum* (wheat), *Alternaria alternate* (apple), *Colletotrichum gloeosporioides* (avocado), *Pgt* (wheat) (Table 6). The homology of five selected CDSs in PHI-base blast was as follows: (i) c55520_g1_i2 with acetyl-CoA acetyltransferase (*Magnaporthe oryzae*), (ii) c86066_g1_i1 with a protein regulating T-toxin biosynthesis (*Bipolaris maydis*), (iii) c21850_g1_i2 with fumonisin C-14 C15-hydroxylase (*Fusarium proliferatum*), (iv) c12596_g1_i2 with aspartic protease (*Colletotrichum gloeosporioides*), and (v) c13574_g1_i1 with putative tryptophan 2 monooxygenase (*Pgt*). These proteins are known to enhance virulence of the respective pathogens. Increased virulence is also anticipated for putative RXLR effector gene (*Phytophthora infestans*) with homology to c45137_g1_i1 and for putative transcription factor, *Amr1* (*A. brassicicola*) with homology to c10109_g1_i2. PHI-base BLAST results revealed maximum homology of *Pt* sequence c13574_g1_i1 with putative tryptophan 2 monooxygenase gene of *Pgt*, which is predicted to increase pathogen virulence.

**Table 6:** PHI BLAST summary of *P. triticina* coding sequences with significant homology to known fungal putative effectors.

Sequence ID	PHI-base entry	Pathogen	Host	Gene/role	Role in host-pathogen interaction	Identity	*E* value
c45137_g1_i1	PHI:4203	*Phytophthora infestans*	*Nicotiana benthamiana*	Putative RXLR effector genes	Increased virulence	24.46	0.00387
c55520_g1_i2	PHI:5082	*Magnaporthe oryzae*	*Oryza sativa*	Acetyl-CoA acetyltransferase	Increased virulence	53.28	4.4E-145
c86066_g1_i1	PHI:2315	*Bipolaris maydis*	*Zea mays*	Regulate T-toxin biosyn thesis	Increased virulence	27.05	0.00984
c21850_g1_i2	PHI:9266	*Fusarium proliferatum*	*Oryza sativa*	Fumonisin C-14, C15-hydroxylase	Increased virulence	32.26	1.46E-20
c12139_g1_i1	PHI: 459	*Ustilago hordei*	*Hordeum vulgare*	Mating locus protein	Loss of pathogenicity	41.38	1.65
c10109_g1_i2	PHI: 2506	*Alternaria brassicicola*	*Brassica oleracea*	Putative transcription factor	Reduced virulence	43.33	0.322
c40637_g1_i2	PHI: 1264	*Fusarium graminearum*	*Triticum* sp	Protein kinase	Increased pathogenicity	32.00	7.19
c3093_g1_i2	PHI: 2400	*Fusarium graminearum*	*Triticum* sp	Toxin Synthesis. pathogenicity and Reproduction	Increased virulence	54.35	9.4E-27
c14094_g1_i1	PHI: 685	*Magnaporthe oryzae*	*Oryza sativa*	Melanin biosynthesis	Loss of pathogenicity	37.93	8.38
c48436_g1_i1	PHI: 160	*Alternaria alternata*	*Malus domestica*	AM toxin synthase	Increased pathogenicity	37.50	1.12
c12596_g1_i2	PHI: 3973	*Colletotrichum gloeosporioides*	*Persea americana*	Aspartic protease	Increased virulence	28.53	2.42E-29
c43030_g2_i1	PHI: 1247	*Fusarium graminearum*	*Triticum* sp.	Protein kinase	Increased pathogenicity	36.11	0.292
c13574_g1_i1	PHI: 3998	*Puccinia graminis*	*Triticum aestivum*	Putative tryptophan 2 monooxygenase	Increased virulence	34.15	1.72

### Assessment of 3D structures

**Figure 4:**
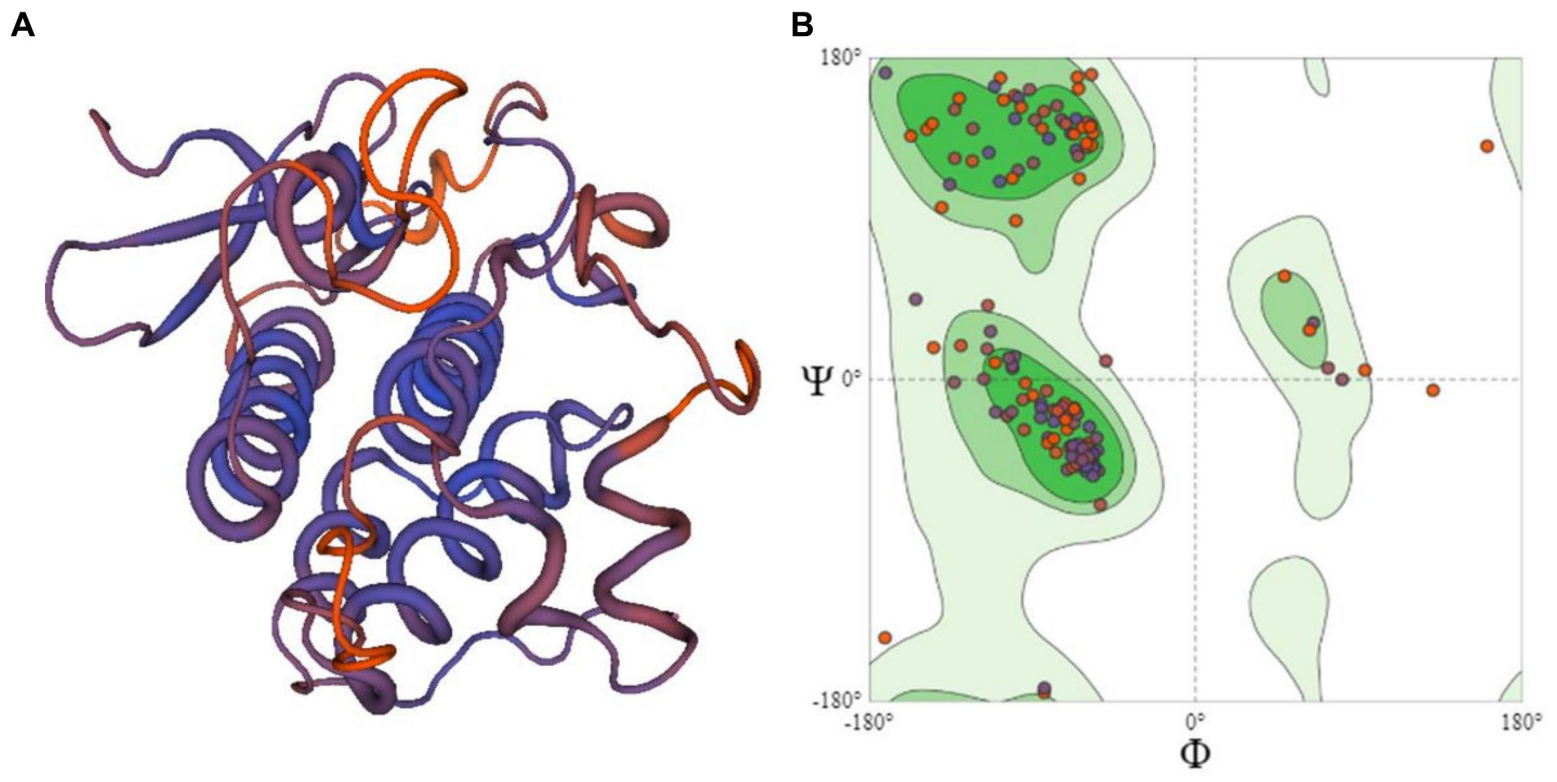
**(A)** The 3D structural model of R protein (PDB id:6cth.1.A) prepared using homology modeling **(B)** Ramachandran plot showing the residues of R protein.

The 3D structure of six candidate effectors (Supplementary Figure S1) and R protein (Figure 4A) was retrieved using homology modeling. R protein residues were in most favored region of 91.1% in accordance with the model generated by SWISS-MODEL structure assessment (Figure 4B). The similarity of 3D structures of all the proteins with corresponding template proteins are given in Supplementary Table S1. Higher GMQE (Global Model Quality Estimation) Score of R protein (0.63) indicated higher reliability of the modeled structures. All effector molecules had a value of 0.7–0.8 except c12596_g1_i2 having 0.1 GMQE value. The phi (ϕ) and psi (φ) torsion angles in Ramachandran plots showed excellent geometry of the modeled 3D structures of R proteins (91.1%) and effector molecules (68.8–93.3% in the favored region) (Supplementary Table S1).

### Molecular docking analysis

**Figure 5:**
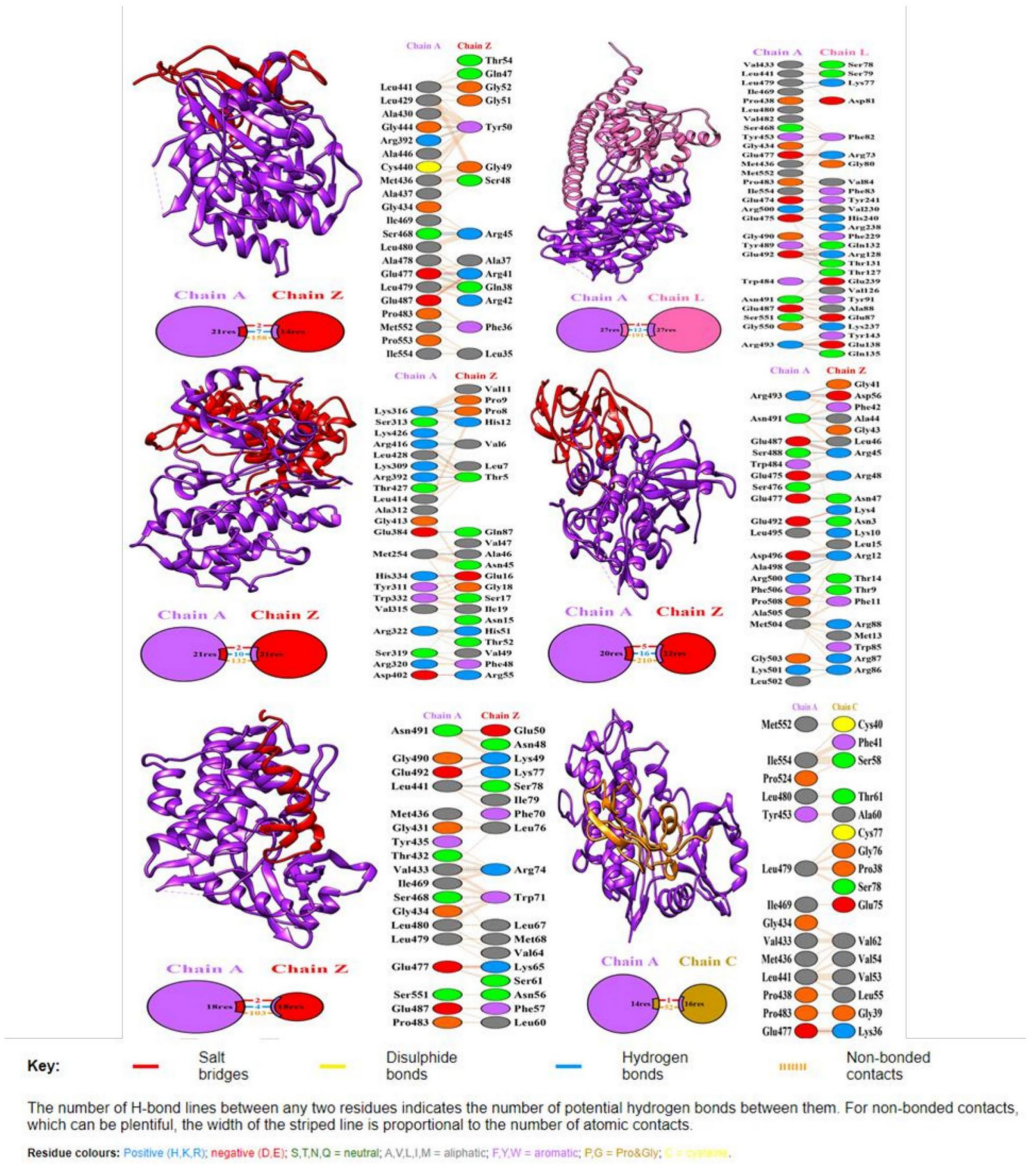
Docking representation of the R protein (Traes_4AL_57B7B4A87) crystal structure (PDB id: 6cth.1.A) and candidate effector complex showing the binding interface of the complex. The interacting chains are joined by colored lines, each representing a different type of interaction. The area of each circle is proportional to the surface area of the corresponding protein chain. The extent of the interface region on each chain is represented by a colored wedge whose color corresponds to the color of the other chain and whose size signifies the interface surface area. Interaction representation including hydrogen, salt bridges, and nonbonded interactions with interface statistics. Chain A is of R protein and chain Z, L, and C of effector molecules.

The Clus-Pro score decreased from a maximum of-858.2 (c14094_g1_i1) to a minimum of −738.9 kJ/mol (c48436_g1_i1), in the R variant complexes. It appears that R protein has a higher preference for the (c14094_g1_i1) variant compared to other effectors (Table 7). The prodigy results for the ΔG calculation with more negative values indicated that R protein receptor molecule binds effectively to effector molecule spontaneously. ΔG for effector c10109_g1_i2 shows maximum negative value of −11.7 and c12139_g1_i1 with −11.6, showing their good interaction and feasibility (Table 7). The dissociation constant (KD) inferred from the binding affinity and dissociation constant values, suggest that R protein binds more strongly to effector c14094_g1_i1 (Kd = 9.1e−09) than to other effector molecules. Their compact binding affinity with pocket fitting in the active regions suggest stable conformation of all the protein-effector complexes. The binding interactions between the amino acids are shown in Figure 5.

**Table 7:** Weighted scores, binding affinity (ΔG), and dissociation constant (Kd) of the interaction of *P. triticina* candidate effector with R protein.

Candidate effector	Cluster	Members (docked conformations)	Representatives	Weighted score KJ/mol	ΔG (kcal mol)	Kd (M) at 25.0°C
c12139_g1_i1	0	256	Center	−759.2	−11.6	2.9e-09
			Lowest energy	−839.7		
c10109_g1_i2	0	126	Center	−834.9	−11.7	2.5e-09
			Lowest energy	−834.9		
c40637_g1_i2	0	76	Center	−811.2	−11.2	5.7e-09
			Lowest energy	−959.4		
c14094_g1_i1	0	120	Center	−741.8	−11.0	9.1e-09
			Lowest energy	−914.7		
c12596_g1_i2	0	190	Center	−858.2	−6.1	3.1e-05
			Lowest energy	−1,006		
c48436_g1_i1	0	166	Center	−738.9	−8.9	3.1e-07
			Lowest energy	−883.4		

### MD simulation analysis

#### Root mean square deviation

**Figure 6:**
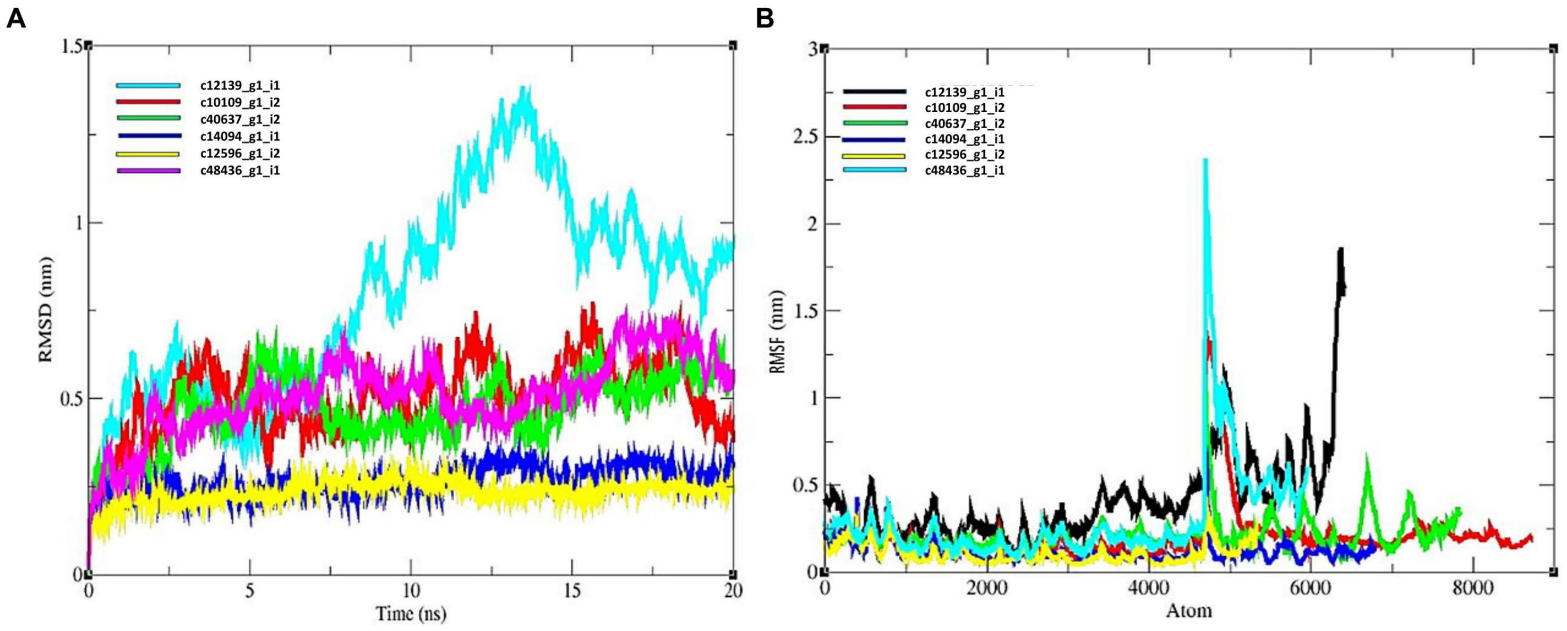
MD simulation results of R protein models with six candidate effectors. **(A)** RMSD plot, and **(B)** RMSF plot.

The average RMSD for candidate effector proteins were as follows: c12139_g1_i1 = 0.8261, c10109_g1_i2 = 0.432, c40637_g1_i2 = 0.531, c14094_g1_i1 = 0.324, c12596_g1_i2 = 0.256, c48436_g1_i1 = 0.589 (Figure 6A). The c12596_g1_i2 displayed low value relative to others. In case of all the predicted hits, R protein and effectors showed a stable complex. All the complexes showed a good score and can be considered for further investigation.

#### Root means square fluctuation

The average value of RMSF for R protein and effector complexes were as follows: c12139_g1_i1 = 0.532, c10109_g1_i2 = 0.235, c40637_g1_i2 = 0.361, c14094_g1_i1 = 0.0238, c12596_g1_i2 = 0. 1,256, c48436_g1_i1 = 0.452 nm, respectively (Figure 6B). Therefore, based on RMSF study, c14094_g1_i1 complex is more stable relative to other protein complexes.

### qRT-PCR expression analysis of candidate effectors

**Figure 7:**
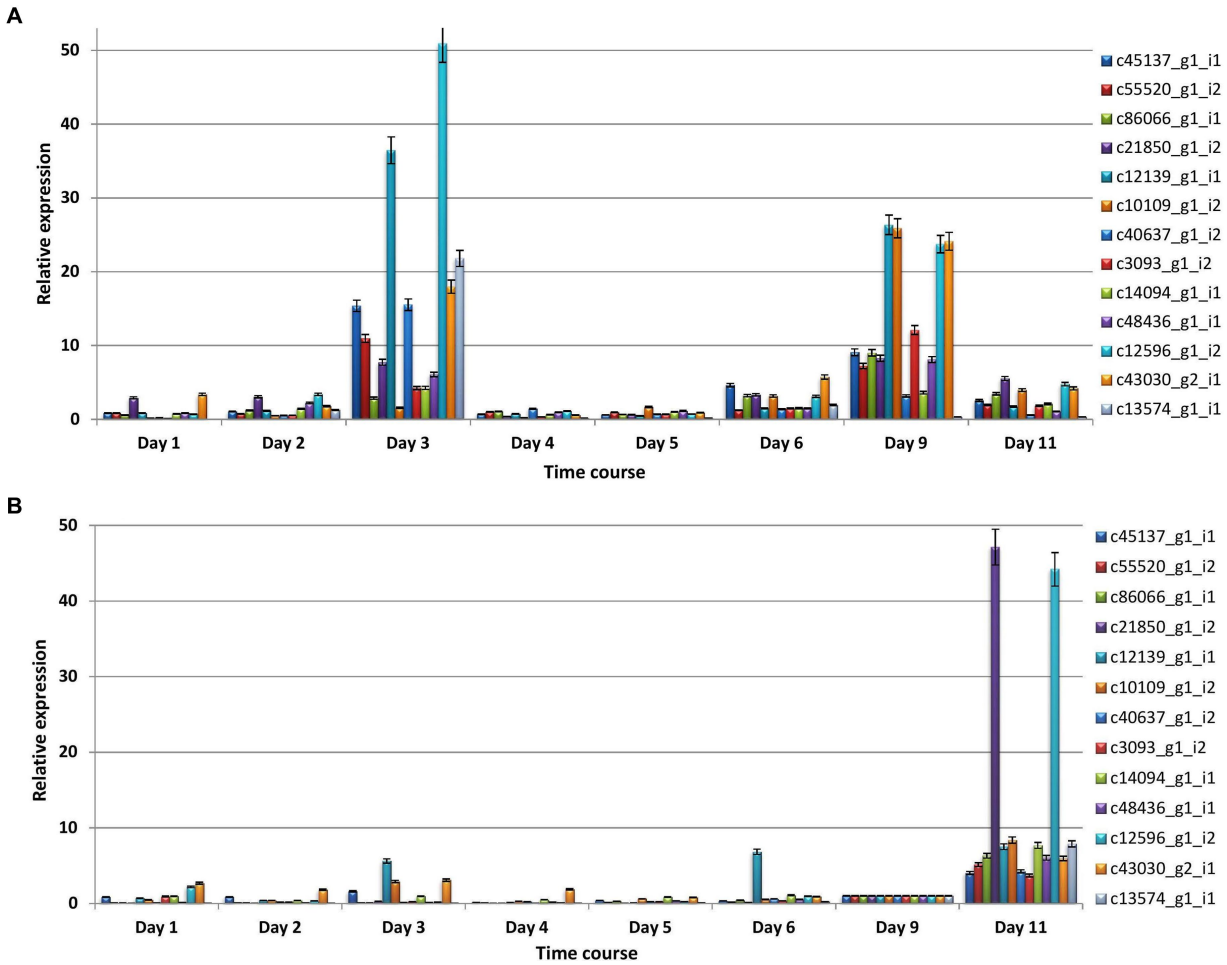
The relative expression profiles of differentially expressed coding sequences of *P. triticina* at different stages (days) after inoculation in a pair of NILs **(A)** HD2329 + *Lr28* (incompatible interaction), and **(B)** HD2329 (compatible interaction).

In majority of cases, relative expression of CDSs increased at 3, 9, and 11 dpi in both compatible and incompatible interactions (Figures 7A,B). The major findings of qRT-PCR analysis include the following.

(i) In incompatible interaction (resistant NIL), the relative expression was maximum at 3 dpi in seven CDSs, at 9 dpi in five sequences; in the remaining one CDS (c21850_gl_i2) maximum expression was observed at 3 and 9 dpi followed by 11 dpi in incompatible interaction (Figure 7A). (ii) In compatible interaction (susceptible NIL), the relative expression of all the CDSs was maximum at 11 dpi, followed by 1, 3, 6, or 9 dpi in that order (Figure 7B).

### Host miRNA interaction with pathogen CDSs

The solitary known miRNA (miR172b) of host had complimentary target sequence to a putative pathogen CDS, c3093_g1_i2 (MW423361) at an expect value of 3.0.

miR172 21 ACGUCGUAGUAGUUCUAAGGG 1 miRNA.

:::::::::::::::::

MW423361 417 ACCAGCAUCAUCAAACUUCCC 437 Pathogen CDS.

The miRNA miR172b was up-regulated at 96 hpi in resistant NIL (R96) in comparison to at 96 hpi in susceptible NIL (S96) during earlier miRNA studies involving the same wheat-rust pathosystem as used during the present study (Jain et al., 2020, 2023).

## Discussion

An understanding of pathogenicity mechanisms of plant biotrophs including wheat rust pathogens is critical for formulating strategies for resistance breeding. It has been suggested that effectors are key pathogenicity factors that determine the nature of host-pathogen interactions, whether compatible or incompatible (Jones and Dangl, 2006; Hogenhout et al., 2009; Prasad et al., 2019). In a recent survey, Louet et al. (2021) examined 249 most highly cited publications focused on plant pathogen effectors, published during 2000–2020 suggesting an increased activity in the area of ‘effectoromics’. In the current study, leaf rust infected wheat transcriptome data was evaluated through multiple bioinformatics analyses to determine the possibility of 13 *Pt* CDSs to be the effector molecules, followed by their expression analysis at different stages of leaf rust disease development under compatible and incompatible interactions. Annotation of these CDSs using five different bioinformatics tools/pipelines (see Section Materials and methods for details) showed potential effector domains in some of the sequences. Further, the results of quantitative Real-time PCR (qRT-PCR) suggested putative effector roles of these sequences.

A BLAST search against NCBI data revealed significant homology between the above CDSs (deposited by us as NCBI Accession numbers MW423354 to MW423366) with some of the previously annotated gene sequences of *Pt* race PtA15 and *Pgt* race 75-36-700-3 (Table 1). The sequences submitted for *Pt* race PtA15 were annotated as predicted proteins, while the virulence role of certain matching proteins found in human and animal fungal pathogens, similar to those annotated in *Pgt* race 75-36-700-3, is well-established and widely known. However, for plant pathogens it is not established in detail, which otherwise might be critical for virulence. Four of these proteins have various role as: (i) stress-induced-phosphoprotein 1 {STIP1, also known as heat shock protein 70/90 (HSP70/90)} is known to regulate different cellular and physiological processes including cell morphology, drug resistance, and virulence in eukaryotes (Singh et al., 2009; Alaalm et al., 2021). (ii) Acetyl-CoA acetyltransferase (encoded by *Pt* homologous CDS c55520_g1_i2), also denoted as ERG10 or Acetoacetyl-CoA thiolase or thiolases, contributes in biosynthesis of isopropanoid that expedites conversion of mevalonate to ergosterol through mevalonate pathway. This is known to have relevance in pathogenesis and virulence, suggesting a positive role of Acetyl-CoA acetyltransferase in facilitating pathogenesis or virulence in pathogenic fungi and bacteria (Gazzerro et al., 2012). The *Pt* sequence c55520_g1_i2 encoding Acetyl-CoA acetyltransferase is also known to have homology with *Magnaporthe oryzae* gene encoding acetyl-CoA C-acetyltransferase in PHI-BLAST. (iii) Like Acetyl-CoA acetyltransferase, the third protein, namely sterol 24-C-methyltransferase (Erg6) is also known to facilitate pathogenicity by contributing in ergosterol biosynthesis through an alternative pathway of ergosterol biosynthesis by converting zymosterol into fecosterol (Nes et al., 2009). (iv) the protein Seryl-tRNA synthetase (ssm1) is another important enzyme that is essential for cellular integrity, viability, and critical to mitochondrial protein synthesis. It helps fungal pathogens to resist host defense mechanisms and indirectly contribute to pathogenicity (Ostrowski and Saville, 2017). Other CDSs had homology with previously annotated (NCBI) ribosomal proteins or hypothetical proteins with unknown functions.

PHI-BLAST tool established homology of *Pt* CDSs with different proteins encoded by genes from different plant pathogens including *P. infestans*, *M. oryzae*, *Fusarium* spp., *U. hordei*, *Pgt, A. alternate*, *C. gloeosporioides*, and others (Table 6). Only some of the above *Pt* genes known to be important factors in avirulence/virulence will be discussed in detail. (i) For instance, the gene *Pgt-IaaM* encoding a putative tryptophan 2-mono-oxygenase, which catalyzes the synthesis of auxin precursor indole-3-acetamide (IAM), has been identified in haustoria of *Pgt* in infected wheat plants, leading to the accumulation of auxin in infected leaf tissue. The indole-3-acetic acid (IAA), generally produced by terrestrial plants, is also reported in some bacterial pathogens, which play important role in pathogenesis (Spaepen and Vanderleyden, 2011). Silencing of *Pgt-IaaM* also indicated that it was required for full pathogenicity during stem rust infection in wheat (Yin et al., 2014). (ii) Melanin, an important pigment in fungi, is utilized by some fungal pathogens like *Colletotrichum lagenarium*, *Magnaporthe grisea* and others for mechanical penetration of host tissue (Howard and Ferrari, 1989). In contrast to the above examples of positive role in pathogenicity, *Amr1* (an important gene contributing to melanin biosynthesis in *Alternaria brassicicola*) has been shown to have a negative effect on pathogen virulence (Cho et al., 2012). (iii) The aspartic proteases (Aps; acid proteases, aspartyl and aspartate proteases) are reported to influence different physiological processes in fungi including pathogenesis and nutrition. The role of transcripts encoding Aps in pathogenicity or virulence have been proved in several plant pathogenic fungi including *Botrytis cinerea*, *Fusarium culmorum*, *Sclerotinia sclerotiorum* and others (Urbanek and Yirdaw, 1984; Poussereau et al., 2001; Have et al., 2004). Conversely, silencing of some of the transcripts encoding APs do not affect pathogenesis in *Botrytis cinerea*, and *Glomerella cingulata* (Have et al., 2004, 2010). Five of the 13 CDSs were annotated as sequences encoding ribosomal proteins in NCBI BLAST. Several ribosomal proteins are reported to induce hypersensitive responses in resistant hosts during incompatible host-pathogen interaction (Friesen et al., 2008). Thus the ribosomal proteins identified during the present study might have some role in avirulence or virulence of the pathogen.

EffectorP, which is known as a potent tool for predicting candidate effectors, revealed the probability of nine *Pt* CDSs being candidate effectors. Among six predicted effector candidate (>0.8 probability) using EffectorP, c14094_g1_i1, c10109_g1_i2, c48436_g1_i1, c40637_g1_i2, and c12139_g1_i1 were annotated as ribosomal proteins in NCBI BLAST, STRING, and InterProScan searches, while the solitary effector candidate c12596_g1_i2 was annotated as ribosomal protein only in NCBI BLAST search and no similarity was found for this sequence in STRING, and InterProScan searches. These identified ribosomal proteins may have a role in virulence/avirulence in biotrophic pathogens such as *Pt* since in an earlier study the ribosomal proteins were reported to function as effectors in necrotrophic fungal plant pathogens (Friesen et al., 2008).

In protein-protein interactions study, the predicted residues revealed the stability of interaction between candidate *Lr28* protein and the effector molecules. The interaction binding energy of most of the molecules was highly negative (−11 kcal/mol) showing a good binding of candidate effectors with R protein as also shown by Sidhu et al. (2020). These results are also in accordance with Comeau et al. (2004), who showed that native folds are typically the cluster with the largest number of low-energy structures and maximum stability.

The molecular dynamics simulation conducted to investigate the binding stability of R protein and effector complexes revealed that all six candidate effectors used for homology modeling exhibited stable RMSD values ranging up to 0.5 nm over 20 ns, although a peak RMSD value of 1.4 nm was observed for effector c12139_g1_i1, which could be due to some structural fluctuations or conformational changes. The level of structural stability suggests that the protein conformation remained relatively stable during the simulation, and the effector molecules effectively stabilized both protein structures in the binding pocket. The majority of protein backbone atoms showed acceptable RMSF values of 0.5 nm. These results and molecular dynamics simulation successfully provided insights into the flexible and stable nature of the interaction between R protein and candidate effector complexes (Gautam et al., 2019; Sidhu et al., 2020).

Few studies have reported the role of host miRNA in targeting pathogen genes during defense (Cai et al., 2018; Li et al., 2021). In a recent study, *in-silico* prediction of a number of pathogen genes targeted by miRNA of the host was carried out in the same wheat-leaf rust pathosystem as investigated during the present study (Shiv et al., 2023). The targeted pathogen genes were involved in different biological processes such as translation initiation, hydrolytic activity and virulence directly or indirectly. In the present study, one such miRNA (miR172b) had potential target in one of the identified pathogen CDS (c3093_g1_i2). Although none of the host miRNA was found to target the six potential candidate effectors identified in this study, however the up-regulation of miRNA (miR172b) in resistance NIL indicates its possible role in targeting and suppressing pathogen gene, c3093_g1_i2. Further validation of role of these miRNAs in targeting pathogen genes that are necessary for growth and sustenance of pathogen needs to be done in future.

Expression profiling of all the 13 CDSs using qRT-PCR suggested that relative expression of all 13 *Pt* CDSs was comparatively more during incompatible interaction than compatible interaction. The expression of *Pt* CDSs was maximum at 3 or 9 dpi in incompatible interaction while in compatible interaction maximum expression of all 13 CDSs was profiled at 11 dpi. These stages in general belong to early infection (3 dpi), sporulation (9 dpi), and proliferation or secondary infection stages (11 dpi) in wheat leaf rust fungus (Cheng et al., 2017; Zhao et al., 2020). The expression of all the CDSs peaked at early infection or sporulation stages in resistant NIL. The genes expressing at these stages are commonly presumed to help in pathogenicity or virulence (Palmiter, 1998). However, in the present study, these genes expressed during early infection or sporulation stages only in incompatible interaction suggesting that the host might have responded to the products of these genes and resulted in resistant response as is the case in effector triggered immunity. An alternative explanation to our observations is that the pathogen might be trying to establish itself and withstand the host’s resistance response subsequent to PAMP triggered immunity. This statement is also evident from the fact that most of the CDSs annotated by us in the NCBI BLAST, such as stress-induced-phosphoprotein 1, acetyl-CoA C-acetyltransferase, sterol 24-C-methyltransferase, and seryl-tRNA synthetase are responsible for different cellular and physiological processes including maintenance of cellular integrity and morphology, strengthening of cell membrane, growth, development, mitochondrial protein synthesis, stress resistance, drug resistance and others (Hsueh et al., 2005; Nes et al., 2009; Gazzerro et al., 2012; Ostrowski and Saville, 2017; Alaalm et al., 2021). The second explanation also supports the induced expression of these genes in compatible interaction at 11 dpi. As discussed earlier, this is a stage when pathogen starts another round of infection cycle. By this stage the level of various nutrients including sugars in the hosts goes down and therefore, the pathogen is stressed for carrying on further growth and development. Consequently, overexpression of these genes might be helping the pathogen to survive under such conditions (Peter et al., 2003; Huang et al., 2011). Based on EffectorP, PREDECTOR, STRING, InterProScan, and qRT-PCR analysis, five CDSs (c14094_g1_i1, c10109_g1_i2, c48436_g1_i1, c40637_g1_i2, and c12139_g1_i1), predicted as ribosomal proteins, and with homology to proteins helping in melanin biosynthesis, putative transcription factor, AM toxin synthase, protein kinase, and mating locus protein, respectively, in PHI-BLAST analysis; and c12596_g1_i2 predicted as hypothetical protein (NCBI BLAST) and with homology to aspartic protease (PHI-BLAST) (Table 6), could be anticipated to have role in pathogen avirulence and temporarily considered as potential *Pt* avirulence determinants (putative effectors). However, further functional characterization of these CDSs would help to critically pinpoint their defined role in pathogen avirulence.

## Conclusion

In the present study, we anticipated the role of 13 CDSs from *Pt* in its avirulence or virulence. The CDSs were annotated and their sequences were deposited in NCBI database, which can be utilized in future. These sequences were further analyzed using EffectorP, PREDECTOR, STRING, InterProScan, and PHI BLAST tools (*in silico*); and qRT-PCR analysis for investigating the possibility of their role as effector candidates. The Molecular docking and MD Simulation analysis based on RMSF study revealed that c14094_g1_i1-candidate *Lr28* protein complex binds more strongly and was more stable than other candidate effectors. The stage specific expression profiles of all CDSs corresponding to *Lr28* suggested that their expression was more pronounced during early infection and sporulation stages in incompatible interactions, which indicate that either they may be acting as avirulence determinants (effectors) or helping the pathogen to survive and withstand host’s immunity responses. The nature of these CDSs/genes corresponding to *Lr28* can be analyzed and validated further, and interaction of their products with the products of *Lr28* (yet to be cloned) could possibly reveal the importance of these genes in wheat-leaf rust interaction and thus assist in developing new strategies for wheat leaf rust management.

## Data availability statement

The datasets presented in this study can be found in online repositories. The names of the repository/repositories and accession number(s) can be found in the article/Supplementary material.

## Author contributions

PG, PP, SB, and HB conceived the experiment. PP, NJ, and SS conducted the *in-silico* analysis. PP, RT, OG, CL, SA, and SK conducted the wet lab experiments and data analysis. JC conducted molecular modeling and simulation studies. PP, PG, SS, and NJ wrote the first draft of the manuscript jointly and finalize it. All authors contributed to the article and approved the submitted version.

## Conflict of interest

The authors declare that the research was conducted in the absence of any commercial or financial relationships that could be construed as a potential conflict of interest.

## Publisher’s note

All claims expressed in this article are solely those of the authors and do not necessarily represent those of their affiliated organizations, or those of the publisher, the editors and the reviewers. Any product that may be evaluated in this article, or claim that may be made by its manufacturer, is not guaranteed or endorsed by the publisher.

## Footnotes

^1^https://www.ncbi.nlm.nih.gov/orffinder; accessed on 08/2021.

^2^http://phi-blast.phi-base.org; accessed on 08/2021.

^3^ https://saves.mbi.ucla.edu/

^4^ https://www.ebi.ac.uk/thornton-srv/software/PROCHECK/

^5^ https://www.doe-mbi.ucla.edu/verify3d/

^6^ https://www.doe-mbi.ucla.edu/errat/

^7^ https://cluspro.bu.edu

^8^ http://www.pymol.org/pymol

^9^ http://www.ebi.ac.uk/thornton-srv/databases/pdbsum/Generate.html

^10^ https://wenmr.science.uu.nl/prodigy/

^11^ https://www.gromacs.org/

^12^ http://plantgrn.noble.org/psRNATarget/
