# Supplementary material for: Candidate effectors for leaf rust resistance gene Lr28 identified through transcriptome and in-silico analysis
Source: Front Microbiol. 2023 Sep 17;14:1143703. doi: 10.3389/fmicb.2023.1143703 (PMC10543267; doi:10.3389/fmicb.2023.1143703)
Supplement: Supplementary file 1 [file Data_Sheet_1.docx]

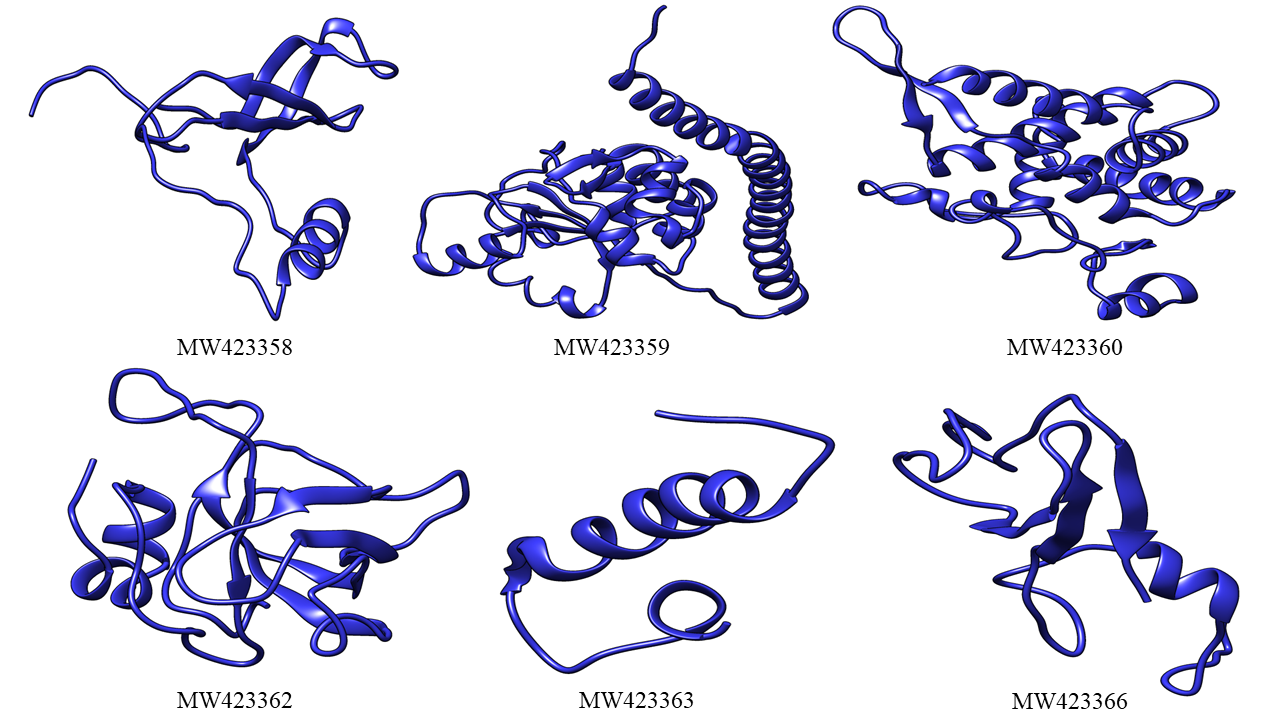
**Supplementary Figure 1.** The 3D structures of 6 candidate effector proteins {c12139_g1_i1 (MW423358), c10109_g1_i2 (MW423359), c40637_g1_i2 (MW423360), c14094_g1_i1 (MW423362), c12596_g1_i2 (MW423363), c48436_g1_i1 (MW423366)}. All the proteins have monomeric structure, where spirals represent helices, broad strips with arrow-head represent β-pleated sheets and thin loops represent coil.

**Supplementary Table S1. 3D structure analysis of Effector and Lr28 proteins used for protein modelling**

| **S.N.** | **Proteins** | **GMQE** | **Q-mean** | **Seq identity %** | **PROCHEK** | | | | | **ERRAT** | **VERIFY 3D** | **Status of 3D structure from SAVES** |
| --- | --- | --- | --- | --- | --- | --- | --- | --- | --- | --- | --- | --- |
|  |  |  |  |  | **Favoured region (%)** | **Allowed region (%)** | **Generously allowed region** | **Disallowed region (%)** | **G Factor** | **Quality factor** | **(3D-1D Profile) (%)** |  |
| 1 | *Lr 28* | 0.63 | 0.68 | 40.3 | 91.1 | 8.5 | 0.4 | 0 | 0.08 | 96.15 | 80 | pass |
| 2 | c12139_g1_i1 | 0.78 | 0.72 | 83.9 | 93.3 | 6.7 | 0 | 0 | -0.02 | 98.1 | 67.9 | Fail |
| 3 | c10109_g1_i2 | 0.83 | 0.8 | 60.8 | 91.6 | 7.5 | 0.5 | 0.5 | -0.03 | 91.2 | 63.1 | Fail |
| 4 | c40637_g1_i2 | 0.79 | 0.73 | 79.3 | 88.6 | 11.4 | 0 | 0 | -0.05 | 89.5 | 39.9 | Fail |
| 5 | c14094_g1_i1 | 0.84 | 0.8 | 81.6 | 93 | 6.1 | 0 | 0.9 | -0.1 | 95.5 | 53.6 | Fail |
| 6 | c12596_g1_i2 | 0.14 | 0.35 | 21 | 68.8 | 28.1 | 3.1 | 0 | -0.2 | 89.6 | 0 | Fail |
| 7 | c48436_g1_i1 | 0.77 | 0.7 | 84.3 | 84.5 | 14.1 | 1.4 | 0 | -0.1 | 67.6 | 21.9 | Fail |
